# AI-enabled and comparator digital interventions for adolescent mental health (2020–2025): early detection, human–AI collaboration, and ethical governance—a PRISMA-ScR scoping review

**DOI:** 10.3389/frcha.2026.1905782

**Published:** 2026-09-30

**Authors:** Noorayisahbe Mohd Yaacob, Mohamed Doheir, Ali Algumaei, Mohammed Nasser Al-Andoli, Jamil Abedalrahim Jamil Alsayaydeh, Abdelrafe Elzamly, Abd Samad Basari

**Affiliations:** 1 Strategic Information and Software Systems (SISS) Research Lab, Center for Software Technology and Management (SOFTAM), Faculty of Information Science and Technology, Universiti Kebangsaan Malaysia (UKM), Selangor, Malaysia; 2 Faculty of Technology Management and Technopreneurship, Universiti Teknikal Malaysia Melaka (UTeM), Melaka, Malaysia; 3 Faculty of Computing and Informatics, Multimedia University, Cyberjaya, Malaysia; 4 Department of Engineering Technology, Universiti Teknikal Malaysia Melaka (UTeM), Melaka, Malaysia; 5 Faculty of Computer and Information Technology, Al-Aqsa University, Gaza, Palestine; 6 Faculty of Computer Science and Information Technology, Universiti Tun Hussein Onn Malaysia (UTHM), Johor, Malaysia

**Keywords:** adolescent mental health, AI chatbots, cultural adaptation, digital mental health interventions, early detection, ethical governance, human–AI collaboration, PRISMA-ScR scoping review

## Abstract

**Introduction:**

Mental health difficulties among adolescents and youth (ages 10–24) are increasing globally, yet early intervention remains impeded by stigma, service shortages, and cultural barriers. AI-enabled technologies including chatbots, automated screening tools, and machine learning-based predictive models have been proposed as scalable approaches to address these gaps, alongside non-AI digital interventions such as therapist-guided internet-delivered cognitive behavioral therapy (iCBT) and smartphone-based CBT applications, which are examined as comparator evidence given their stronger controlled-design evidence base.

**Methods:**

This scoping review followed Preferred Reporting Items for Systematic Reviews and Meta-Analyses extension for Scoping Reviews (PRISMA-ScR) reporting guidelines and Joanna Briggs Institute (JBI) methodology. A systematic database search across six databases (PubMed, Scopus, ProQuest, SpringerLink, Web of Science, and ScienceDirect), supplemented by manual and forward-citation searching, yielded 10,962 records, of which 19 studies met the predefined inclusion criteria. A Human-AI Collaboration Role framework comprising a four-level continuum from fully human-led to fully automated delivery was applied as a predefined coding dimension across all included studies.

**Results:**

Most studies focused on depression and anxiety among mid-to-late adolescents in high-income countries (68.4%). Therapist-guided iCBT was the most rigorously studied format using randomized and controlled designs; AI chatbots, predictive models, and large language model (LLM)-based systems demonstrated feasibility or engagement outcomes in a limited number of studies and contexts, though randomized diagnostic chatbot trials specifically remain absent for participants under 18 years; at least one randomized non-diagnostic chatbot trial (a body-image intervention) has been conducted in this age group. Equity-related reporting was inconsistent: socioeconomic status was reported in only 10.5% of studies and cultural adaptation in 42.1%; restriction to English-language publications may further underrepresent non-Western and low- and middle-income-country evidence relevant to these equity findings. Key implementation challenges included inconsistent safeguarding protocols for minors, algorithmic bias, data privacy concerns, and insufficient integration with clinical and educational systems.

**Discussion:**

The evidence base remains preliminary and methodologically heterogeneous, insufficient to support conclusions about clinical effectiveness or generalizability to diverse adolescent populations. These findings represent a descriptive map of the current evidence landscape rather than an assessment of effectiveness. Future research should prioritize adequately powered adolescent-focused trials, participatory co-design approaches, culturally informed adaptations, and transparent ethical governance frameworks to ensure responsible development of AI-enabled digital mental health tools for youth. Because the included studies were heterogeneous and no formal risk-of-bias assessment was undertaken, this review does not establish comparative clinical effectiveness, safety, or superiority of AI-enabled interventions.

**Systematic Review Registration:**

https://osf.io/sbpk2, doi: 10.17605/OSF.IO/SBPK2.

## 1 Introduction

Mental health disorders among the younger generation are significant public health concerns. As suggested by the World Mental Health Report presented by the World Health Organization (WHO) in 2022, 8% of those aged 5–9 and 14% of those aged 10–19 are affected by mental health disorders across the world (1). Mental health disorders are one of the most significant causes of Years Lived with Disability and are also key to the current social and economic burden across the world (1). The COVID-19 pandemic further accelerated depression and anxiety rates among the younger population worldwide (2). Unipolar depression is anticipated to become the key contributor to the global disease burden by 2030 (2).

These concerns are not confined to high-income countries. Evidence from low- and middle-income countries (LMICs) shows a parallel rise in mental health conditions accompanied by high out-of-pocket expenditures, workforce shortages, and systemic barriers to care (3). In Malaysia, population data indicate significant psychological distress caused by stigma, poor mental health literacy, and the lack of accessible services (4, 5).

Adolescence and young adulthood are critical periods during which depression and elevated levels of stress and anxiety commonly emerge (6–9). Early depression can interfere with education and interpersonal relationships in ways that persist throughout life (10–13). Early depression is difficult to diagnose because subthreshold symptoms are often overlooked during normal consultations due to limited time to evaluate adolescents properly (14–17).

Digital Mental Health (DMH) interventions are promising approaches to mitigate such structural and psychosocial challenges. Online and application-based platforms offer scalable approaches to increasing reach, reducing access costs, and providing pathways that may help reduce help-seeking stigma (18–21). Online CBT with therapist guidance and smartphone-based CBT applications are widely used in the management of adolescents with depression and anxiety and are increasingly supported within stepped-care approaches (5, 22–24). Despite this potential, current DMH platforms face significant challenges including limited personalization, cultural incompatibility, and ethical concerns around data privacy and governance (25).

The use of artificial intelligence (AI) combined with advances in natural language processing has driven development of conversational agents capable of delivering standardized assessments, psychoeducation, and indirect support (26–28). Foundational reviews of mental health chatbot features (26, 29) provide the conceptual basis for the taxonomy applied in this review and are cited for background framing; both predate or extend beyond the 2020–2025 inclusion window and are not part of the included corpus. Key challenges that still require addressing include potential lack of adequate validation testing, poor prolonged use rates, possible systematic algorithmic biases, and possible adverse experiences in vulnerable end-users (30). Recent adaptable chatbots targeting East and Southeast Asian contexts have shown positive shifts in user engagement and cultural relevance (31, 32). Despite rapid expansion of digital mental health technologies, youth-specific AI validation, ethical governance, and culturally adaptive deployment remain underdeveloped. Four of the present authors previously published a related systematic review of AI-based mental health chatbots covering the 2020–2025 period (27).

The earlier review synthesized 25 peer-reviewed studies across a broad, all-age mental health chatbot literature, with primary analytical emphasis on therapeutic models, technical and system design, cultural adaptation, and ethical considerations. The two reviews also share a subset of primary studies, including Daley et al. (33), de Graff et al. (34), Jiang and Yang (35), and Liu et al. (36). In contrast, the present review was designed as a youth-focused scoping review covering participants aged 10–24 years and addresses a different set of mapping questions concerning early detection, AI-enabled intervention types, human–AI role distribution, equity reporting, and ethical governance. The present review therefore provides a youth-specific evidence map that was not provided by the earlier all-age review, despite the acknowledged overlap in a subset of primary studies. We further clarify the relationship between the two reviews’ analytic devices: the earlier review introduced “symmetry” as a thematic, descriptive lens encompassing cultural, therapeutic, ethical, and structural dimensions, the last of which the earlier paper labeled “Structural Symmetry” applied qualitatively to characterize design patterns across its 25 included systems, without an operationalized classification scheme. The present review’s protocol working title, “Human–AI Symmetry,” extended this thematic language to the specific question of balance between human and AI responsibility in care delivery, and was subsequently operationalized into the discrete four-level coding scheme presented in Table 1 and applied systematically to classify all 19 included studies a level of operational specificity absent from the earlier paper’s discursive treatment of symmetry. It was relabeled “Human–AI Collaboration Role” during manuscript preparation to reflect this operational, rather than thematic, character. The two frameworks share a common thematic origin but are operationally distinct. These gaps motivate the present review, which maps AI-enabled youth digital mental health interventions (2020–2025) across technological, clinical, and ethical dimensions.

**Table 1:** Operational definitions used in this review.

Term	Definition used
AI chatbot	A conversational software agent using rule-based logic, machine learning, or large language models to generate responses
Digital mental health intervention	A technology-mediated tool supporting mental health screening, prevention, or management
Early detection	AI-assisted identification of symptom patterns or risk signals prior to formal clinical diagnosis
Youth	Individuals aged 10–24 years according to WHO classification
Adolescent	Individuals aged 10–19 years, consistent with WHO classification; used in this review to refer specifically to the sub-population under 18 years unless otherwise stated
Young adult	Individuals aged 18–24 years; used in this review to distinguish participants who have reached the age of legal majority in most jurisdictions from those who have not
Minor	Individuals under 18 years of age; used specifically in discussions of consent, safeguarding, and legal applicability
Human–AI collaboration role	The functional division of responsibility between automated AI system outputs and human involvement (clinician, therapist, caregiver, or educator) in decision-making, oversight, and care delivery. Operationalized as a four-level continuum: (1) No AI human-led digital delivery; (2) AI-assisted AI supplements human decision-making; (3) AI-primary AI leads interaction with human escalation pathway available; (4) Fully automated human involvement explicitly absent or the system is explicitly described as operating without human oversight.

This scoping review addresses these gaps by systematically mapping digital interventions for adolescent and youth mental health (10–24 years), with primary emphasis on AI-driven chatbots, automated screening tools, and machine learning-based predictive models for early detection and diagnostic support. Non-AI digital interventions including therapist-guided iCBT and smartphone-based CBT are included as methodologically rigorous comparators that contextualize the AI evidence base. A Human–AI Collaboration Role framework provides the analytic structure through which intervention categories are distinguished and mapped (Section 2). A scoping review was adopted to map the breadth of evidence rather than to synthesize effect sizes. This review synthesizes studies published between 2020 and 2025 across five domains: (i) demographic and contextual characteristics; (ii) targeted mental health domains; (iii) distribution of coded themes within the mapped corpus; (iv) types, mechanisms, and characteristics of digital and AI-based interventions; and (v) ethical, cultural, and implementation challenges. Existing reviews have already synthesized important aspects of youth mental health conversational agents and AI-enabled digital interventions. Balan et al. (37) examined automated conversational agents for young people, while Park et al. (38) reviewed 39 studies of mental health conversational agents for youth. Madera et al. subsequently mapped 56 studies of conversational agents among adolescents aged 10–25 years, focusing on mental health, well-being, and social inclusion (39). Feng et al. (40) conducted a systematic review and meta-analysis of 15 randomized controlled trials involving young people aged 12–25 years, focusing specifically on the effectiveness of AI-driven conversational agents for mental health outcomes.

The present review therefore does not claim chronological novelty. Instead, it extends this literature through a broader youth-focused evidence map covering participants aged 10–24 years and integrating early detection and diagnostic-support applications, AI-enabled intervention types, non-AI digital interventions as methodological comparators, Human–AI Collaboration Roles, demographic and equity reporting, and ethical, cultural, and implementation considerations. Unlike effectiveness-focused meta-analyses restricted to randomized trials, the present scoping review maps the breadth and characteristics of the evidence rather than estimating pooled effects. Its Human–AI Collaboration Role framework is applied as a systematic coding dimension across the included studies to characterize the distribution of human and AI responsibilities. The review was set to integrate existing evidence on digital mental health interventions among adolescents and younger adults with emphasis on AI-based chatbots useful in the early detection process. Five research questions framed the review: (RQ1) which demographic subgroups are most frequently represented in digital mental health research from 2020 to 2025; (RQ2) which mental health domains are most frequently addressed; (RQ3) how are coded themes distributed within the mapped corpus across 2020–2025; (RQ4) what types of digital interventions, particularly AI-based chatbots, have been explored to support adolescent mental health; and (RQ5) what methodological and ethical limitations are reported in studies employing AI-based tools for early mental health screening or diagnostic support. Together, these questions capture the demographic, clinical, technological, and ethical dimensions of the field, while identifying persistent methodological gaps.

## 2 Materials and methods

This scoping review followed the Preferred Reporting Items for Systematic Reviews and Meta-Analyses extension for Scoping Reviews (PRISMA-ScR) reporting guidance (41) and Joanna Briggs Institute (JBI) methodological recommendations (42), using the Population Concept Context (PCC) framework. A three-step search strategy and standardized procedures for screening and data charting were applied.

### 2.1 Review design

The review followed the PRISMA-ScR guidelines (41) and was methodologically aligned with the JBI framework (42, 43). The intention of this review is to map the extent, design considerations, and ethical aspects of digital and AI-powered mental health interventions targeting individuals aged 10–24 years. The review covered the period from 2020 to 2025, consistent with recommendations for scoping reviews addressing emerging and heterogeneous evidence bases (44, 45). The protocol for this scoping review was created on the Open Science Framework (OSF) on 18 October 2025, prior to the November 2025 database search, at: https://osf.io/z3usf. The protocol was subsequently frozen as a formally registered, DOI-assigned record (https://osf.io/sbpk2; DOI: 10.17605/OSF.IO/SBPK2) on 11 March 2026. Eligibility criteria and the coding framework were finalized prior to full-text screening; we note for transparency that the formal registration record was frozen after the initial database search, and we describe this timeline explicitly here in the interest of full methodological transparency, conducted in accordance with PRISMA-ScR and JBI methodological guidance (45).

In line with PRISMA-ScR guidance, no formal risk-of-bias or methodological quality assessment was performed. The inclusion of diverse study designs reflects the emerging and interdisciplinary nature of AI-based digital mental health research. These studies are presented for mapping purposes only and do not imply equivalent levels of evidence or methodological strength.

To ensure coherence across all methodological components, the alignment between the review question, eligibility criteria, included studies, and synthesis framework was explicitly verified prior to data charting. Included studies were organized into five evidence categories defined a priori: intervention and feasibility studies, AI screening and validation studies, user-perception studies, protocols, and conceptual analyses. The synthesis framework maps each included study across the five research question domains using descriptive statistics and thematic categorization. The Human–AI Collaboration Role framework was applied as a predefined coding dimension to all 19 included studies, as defined in Table 1.

### 2.2 Operational definitions

For consistency during screening and data charting, key constructs were operationally defined prior to full review (Table 1).

The four-level continuum draws on prior frameworks distinguishing levels of automation and human oversight in AI-assisted clinical delivery (26, 29), adapted here to educational, clinical, and community deployment contexts relevant to adolescent mental health. This framework was developed for the present review under the working title “Human–AI Symmetry” during protocol registration and was subsequently relabeled “Human–AI Collaboration Role” during manuscript preparation to more precisely describe its functional (rather than structural) analytic focus; the underlying four-level coding scheme is unchanged from the registered protocol. The construct was established prior to data charting and applied as a predefined coding dimension to all 19 included studies, as defined in Table 1. Early detection and intervention functions were further operationalized into three categories (Table 2).

**Table 2:** Operational definitions of early detection and intervention functions in AI-based mental health systems.

Function category	Operational definition
Intervention support	Digital delivery of therapeutic content or guidance, including CBT-based exercises, psychoeducation, conversational support, or behavioral prompts aimed at symptom management.
Monitoring	Continuous or periodic tracking of user behavior, mood, language, or engagement patterns to identify changes in mental health status over time.
Referral and escalation	Automated or semi-automated mechanisms that prompt referral to clinicians, caregivers, or emergency services based on predefined risk thresholds.

### 2.3 Eligibility criteria

Predefined inclusion and exclusion criteria were adopted to ensure objectivity and relevance (Table 3). Included studies concerned digital or AI-based interventions focusing on individuals aged 24 years or below, with sufficient methodological detail for assessment. Protocols and conceptual/system-level papers were retained where they directly informed early detection, ethical governance, or implementation maturity.

**Table 3:** Inclusion and exclusion criteria.

Inclusion criteria (IC)	Exclusion criteria (EC)
IC1: Peer-reviewed journal articles (empirical studies, protocols, and conceptual/system-level analyses) published between 2020 and 2025.	EC1: Studies focusing on adults only (>24 years) or mixed-age samples where youth data could not be separated.
IC2: Participants aged 24 years or below.	EC2: Non-English publications.
IC3: Digital or AI-enabled tools for mental health screening, assessment, diagnostic support, prevention, or intervention.	EC3: Insufficient methodological reporting or unclear/ambiguous outcomes that prevented interpretation.
IC4: English-language publications.	EC4: Conference abstracts, editorials, letters, commentaries, or records without accessible full text.
IC5: Full-text available with sufficient methodological detail to support data charting.	EC5: Duplicate records and non-retrievable reports after full-text search attempts.

For synthesis purposes, included studies are organized into five evidence categories defined a priori: (1) *Intervention and feasibility studies* RCTs, observational, pre-post, quasi-experimental, and feasibility designs evaluating or preparing digital mental health interventions, including studies with intervention outcomes as well as feasibility or trial-planning evidence (n=11); (2) *AI screening and validation studies* studies developing or validating machine learning-based or LLM-based screening tools against clinical criteria (n=4: Tate2020, Weintraub2023, Levy2023, Liu2025); (3) *User-perception studies* cross-sectional surveys reporting adolescents’ use of, attitudes toward, or emotional reactions to AI tools, without delivering or evaluating a clinical intervention (n=2); (4) *Study protocols* registered protocols for planned trials with no outcome results available (n=1); and (5) *Conceptual or system-level analyses* narrative reviews and governance frameworks contributing no empirical intervention data (n=1: Adesina2024). These five categories are analyzed separately throughout the synthesis.

### 2.4 Search strategy

A comprehensive literature search was conducted to identify studies examining AI-based chatbots and digital interventions in adolescent and youth mental health. Searches were performed across six electronic databases: PubMed, Scopus, ProQuest, SpringerLink, Web of Science, and ScienceDirect. The multi-database strategy reduces the risk of underrepresentation of technical studies and enhances completeness of evidence retrieval.

The search strategy was conceptually structured around four domains: (i) target population (adolescents and young people); (ii) mental health conditions and outcomes; (iii) AI-driven or digital technologies (e.g., chatbots, conversational agents, mobile and web-based interventions); and (iv) clinical purposes including early detection, screening, diagnostic support, and risk assessment. Search terms covered the full spectrum from fully automated to human-led tools. Boolean operators were used to combine conceptual domains Free-text, title/abstract-field searching was used across all six databases, including PubMed, consistent with JBI guidance (42); controlled vocabulary (e.g., Medical Subject Headings [MeSH]) was not applied in the PubMed search as executed, which we note as a methodological limitation. Search syntax was adapted to each database’s technical requirements; for ScienceDirect, truncation wildcards were omitted where not supported by the platform interface.

The conceptual Boolean search frameworks are presented in Table 4. Full database-specific search strategies are provided in Supplementary File S1.

**Table 4:** Conceptual boolean search framework used for literature retrieval.

No.	Conceptual search logic
SS1	Adolescents and youth AND mental health conditions AND AI-based chatbots or conversational agents used for early detection, screening, diagnostic support, risk assessment, or symptom monitoring.
SS2	Adolescents and youth AND digital mental health interventions, including non-AI digital interventions retained as comparator evidence, targeting mental health outcomes with a focus on screening, early identification, or symptom assessment.
SS3	Adolescents and youth AND artificial intelligence or machine learning techniques applied to mental health screening, prediction, clinical decision support, or severity estimation.
SS4	Adolescents and youth AND AI chatbots or generative AI systems in mental health contexts, with explicit attention to ethical, privacy, safety, governance, and risk-management considerations.

Search logic was adapted to database-specific syntax. Searches were limited to English-language publications published between January 2020 and November 2025. The table presents the conceptual search framework rather than a verbatim representation of the executed database syntax. The exact database-specific search strings are reported in Supplementary File S1. Non-AI comparator interventions identified through the supplementary manual and forward citation search are therefore not represented as a separate database-search branch.

The primary database searches were executed in November 2025 and covered literature published from 1 January 2020 through the date of search execution. A supplementary manual search of key journals and forward citation checking was subsequently conducted in January 2026, specifically to capture the November–December 2025 window not yet covered by the primary search. This supplementary step identified no additional eligible studies.

### 2.5 Study selection

Search results from all six databases were retrieved and merged into a single dataset prior to screening. The study selection process followed four sequential stages in accordance with PRISMA-ScR guidance (41).

**Stage 1 Identification.** Database searches yielded a total of 10,962 records: PubMed (n=837), Scopus (n=2,365), Web of Science (n=1,607), SpringerLink (n=2,249), ProQuest (n=2,149), and ScienceDirect (n=1,755).

**Stage 2 Deduplication and initial removal.** A total of 3,600 duplicate records were identified and removed. A further 200 records were removed: non-research materials such as news items, editorials, and opinion pieces (n=84); duplicate exports not detected during automated deduplication (n=67); and incomplete bibliographic records (n=49). This left 7,162 records for title and abstract screening.

**Stage 3 Title and abstract screening.** Two independent reviewers screened all 7,162 records against predefined eligibility criteria. Records were excluded for the following reasons: irrelevance to the study population (n=3,401); absence of digital or AI-based tools (n=1,980); and ineligible study design or record type (n=890). A total of 6,271 records were excluded at this stage, leaving 891 records. Disagreements were resolved through discussion and consensus between the two reviewers. A pilot calibration exercise was conducted on approximately 10% of retrieved records prior to full screening (44).

**Stage 4 Full-text eligibility assessment.** Prior to full-text retrieval, 167 records were excluded: conference abstracts and proceedings (n=74); sources without accessible full texts (n=53); and non-article materials (n=40). The remaining 724 full-text reports were retrieved and assessed for eligibility. Of these, 705 were excluded for the following reasons: not focused on chatbot or AI-based mental health tools as defined in the eligibility criteria (n=321); not focused on adolescent or youth populations, including adult-only or mixed-age samples where youth data could not be separated (n=234); and not peer-reviewed or presenting weak methodological reporting insufficient to support data charting (n=150). Nineteen studies met all predefined inclusion criteria and were included in the final synthesis.

The retention rationale for studies with less direct fit to the primary AI chatbot focus is noted here for transparency. The six primary database searches (Table 4; Supplementary File S1) required an AI- or chatbot-related term in combination with population and mental-health terms across all six databases. Non-AI comparator studies retained in this review, specifically therapist-guided iCBT interventions containing no AI component (23, 46) could not have been identified through this AI-focused search logic because these studies do not report an AI- or chatbot-related term in their titles or abstracts. They were instead identified through a prespecified supplementary identification step using manual searching and forward citation checking in January 2026, which was not restricted to AI-specific terminology. We note this because Table 4’s conceptual search framework (SS2) references non-AI digital interventions as a search target, whereas the database strings as executed in Supplementary File S1 do not include a corresponding non-AI-specific branch; SS2 therefore describes the conceptual scope of the review rather than the literal database syntax executed. Cross-sectional user-perception studies (47, 48) were retained because they provide empirical evidence on how adolescents engage with AI-enabled tools in naturalistic settings. The conceptual governance analysis (49) was retained because it directly addresses ethical governance and safeguarding frameworks for AI-enabled adolescent mental health tools (RQ5). The study protocol (50) was retained because protocols for planned trials are explicitly permitted under IC1. In all cases, retained studies met predefined eligibility criteria and are presented within their designated evidence category, with no clinical outcome conclusions drawn from non-intervention sources. The entire selection process is illustrated in the PRISMA flow diagram (Figure 1).

**Figure 1:**
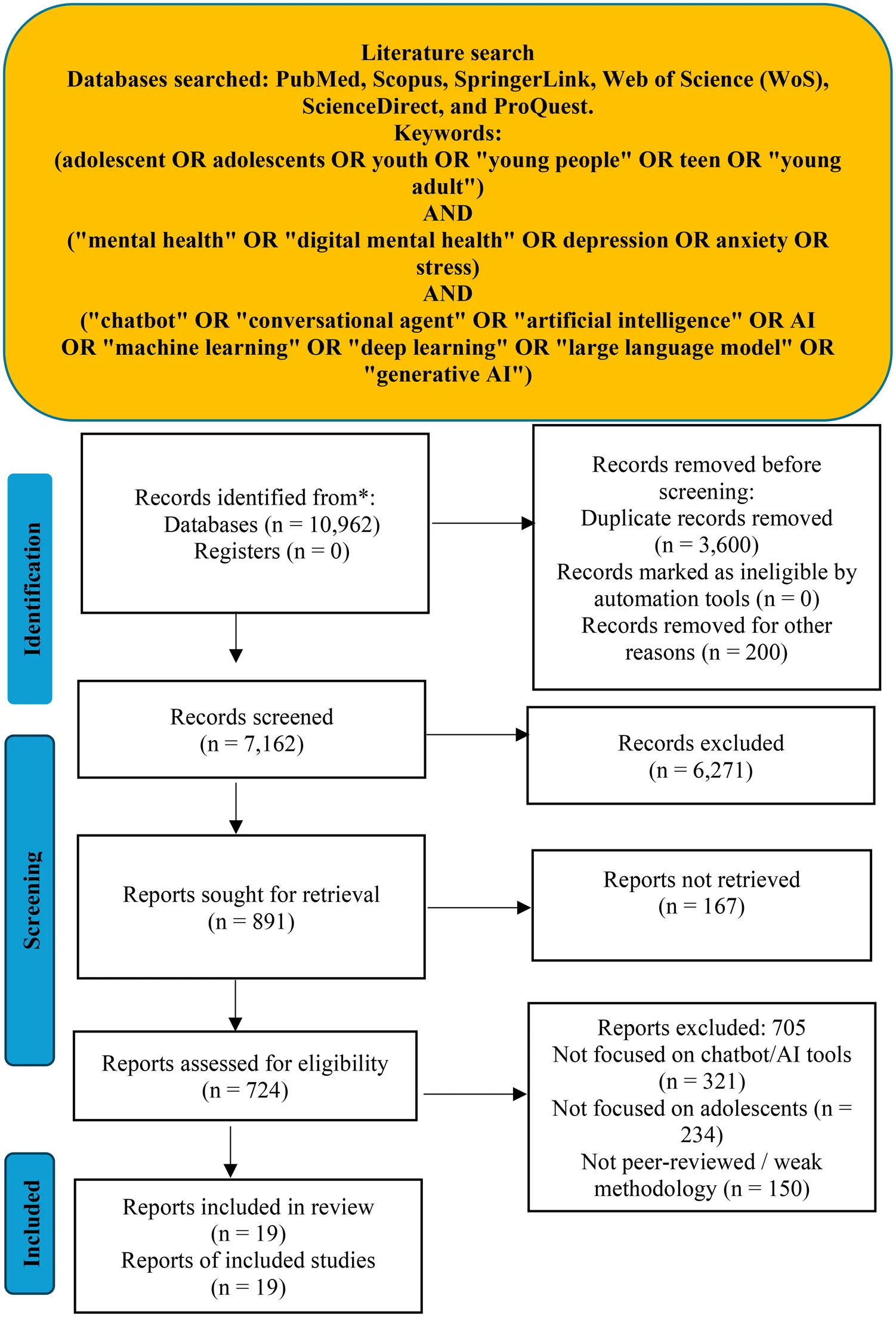
PRISMA-ScR Flow Diagram for Study Selection. Records were identified across six databases: PubMed (n=837), Scopus (n=2,365), Web of Science (n=1,607), SpringerLink (n=2,249), ProQuest (n=2,149), and ScienceDirect (n=1,755); total n=10,962. Database searches were executed in November 2025; a supplementary manual search covering November–December 2025 was conducted in January 2026 and identified no additional eligible studies. After deduplication (n=3,600) and removal of records for other reasons (n=200), 7,162 records proceeded to title and abstract screening. Following systematic screening (6,271 excluded), 891 records remained.Of these, 167 records were not retrieved prior to full-text assessment (conference abstracts and proceedings, n=74; sources without accessible full texts, n=53; non-article materials, n=40), distinct from records that were retrieved but subsequently excluded at full-text review. Of the remaining 724 full texts retrieved and assessed, 705 were excluded at the eligibility stage (not focused on chatbot/AI tools, n=321; not focused on adolescents, n=234; not peer-reviewed or weak methodology, n=150) and 19 studies met all predefined inclusion criteria and were included in the final synthesis.

#### 2.5.1 Inter-rater reliability

Independent screening of titles/abstracts and full-text articles was conducted by two reviewers using a screening guide developed prior to screening to operationalize the eligibility criteria consistently across both stages. Inter-reviewer agreement was high, with percentage agreement of 91% for title and abstract screening and 88% for full-text eligibility assessment. Of the 7,162 records screened at the title and abstract stage, disagreements occurred in approximately 9% of cases. All disagreements were resolved through discussion and consensus between the two reviewers. We acknowledge that chance-corrected measures such as Cohen’s kappa may provide a more robust estimate of inter-reviewer reliability, and the absence of kappa is recognized as a methodological limitation.

### 2.6 Data extraction and calibration

A standardized data-extraction framework (Microsoft Excel) was developed, consistent with scoping review charting guidance (42, 43), to ensure consistency and replicability. Extracted variables included: bibliographic details (author, year, country, journal); study design, sample characteristics, setting, and income level; intervention type and delivery mode; outcomes (symptom scales, engagement, diagnostic accuracy); equity variables (gender, socioeconomic status, ethnicity, adaptation); and ethical and implementation notes.

Data extraction was performed by two independent researchers. A pilot calibration on 10% of the eligible studies was conducted to ensure consistency, with further adjudication by a third researcher where needed. The data-charting form was developed iteratively and refined after piloting to ensure clarity, completeness, and consistency. During data charting, tools were examined not only for therapeutic content but also for their capability to identify emerging symptoms, risk markers, or behavioral changes. Studies were therefore coded according to whether they included predictive screening, symptom monitoring, triage decision support, or passive data-driven risk modeling. Where necessary, corresponding authors were contacted to obtain missing data and verify study details. In light of an extraction inaccuracy identified in a related prior review by the authors (a source study’s general-population sample mischaracterized as adolescent-specific), the population and design characteristics of all 19 studies included in the present review were independently re-verified against their original source publications during data charting.

### 2.7 Data synthesis and analysis

Data integration required descriptive statistics and thematic content analysis, conducted in accordance with narrative synthesis guidance (51), organizing findings thematically across the five research question domains using descriptive statistics and textual summary. Quantitative data (year of publication, geographical distribution, mental health domain coverage) were displayed in frequency and percentage distributions. Thematic data were coded inductively to derive categories including intervention types, technology design, mental health topics, and ethics. No formal critical appraisal or risk-of-bias assessment of included studies was conducted, consistent with scoping review methodology (41, 45).

An additional conceptual synthesis layer was applied: each included study was categorized according to (i) AI function (screening, conversational support, monitoring, or hybrid); (ii) level of clinical integration (standalone, therapist-assisted, or system-integrated); and (iii) stage of technological maturity (feasibility, pilot efficacy, clinical validation, or implementation research). The maturity continuum is a descriptive mapping tool derived from reported study designs and authors’ stated objectives. It does not constitute an evaluative judgment about the quality or methodological strength of any individual study.

## 3 Results

A total of 10,962 records were identified across six databases. After removing 3,600 duplicate records and 200 records for other reasons, 7,162 titles and abstracts were screened. Of these, 891 records remained after title and abstract screening, of which 724 full-text reports were assessed for eligibility. Ultimately, 19 studies (2020–2025) met all inclusion criteria. Study selection followed PRISMA-ScR guidelines (Figure 1).

It is important to distinguish between the number of included studies reported in the PRISMA flow diagram (n=19; Figure 1) and the total number of references cited in the manuscript. The latter includes additional sources used for background context, methodological guidance, and discussion, which are not part of the final included study set.

The 19 included studies comprised heterogeneous evidence types distributed across the 2020–2025 period. Because the corpus is small, these distributions are presented descriptively and are not interpreted as evidence of a publication trend. The 19 included studies represent substantively different types of evidence, organized into five categories as defined in Section 2: (1) intervention and feasibility studies (n=11); (2) AI screening and validation studies (n=4); (3) user-perception studies (n=2); (4) a study protocol (n=1); and (5) conceptual or system-level analyses (n=1).

### 3.1 RQ1: demographic characteristics

Demographic, geographic, and contextual characteristics are summarized in Supplementary Tables S1 and S2. Most studies focused on mid-to-late adolescents, with limited inclusion of early adolescents (52). Research settings included schools, Child and Adolescent Mental Health Services (CAMHS), primary care, community environments, and fully remote platforms (23, 53, 54). AI applications spanned therapeutic chatbots, CBT apps, predictive machine learning (ML) models, and LLM-based conversational systems (36, 55).

#### 3.1.1 Age subgroup distribution

Because the 10–24 year youth population spans children, adolescents, and young adults, we classified each included study by whether its enrolled age range fell exclusively under 18 years, exclusively 18 years or older, or spanned both categories, based on the age ranges reported in Supplementary Table S1. Of the 19 included studies, 4 enrolled participants exclusively under 18 years (23, 52, 56, 57), 3 enrolled participants exclusively 18 years or older (33, 34, 36), and 9 enrolled samples spanning both age categories (35, 46–48, 50, 53, 54, 58, 59). Age range was reported only in broad descriptive terms for 2 studies (55, 60), and age was not applicable for the single conceptual governance paper (49). This age distribution is directly relevant to questions of consent, safeguarding, and legal applicability discussed in Section 4.7 below.

#### 3.1.2 Geographic and economic distribution

Research was concentrated in high-income countries (HICs) (68.4%), with emerging contributions from upper-middle-income countries(UMICs) and LMICs (33, 34). Distribution across World Bank income groups is shown in Figure 2 (Table 5). Country-level distribution of included studies is shown in Figure 3.

**Figure 2:**
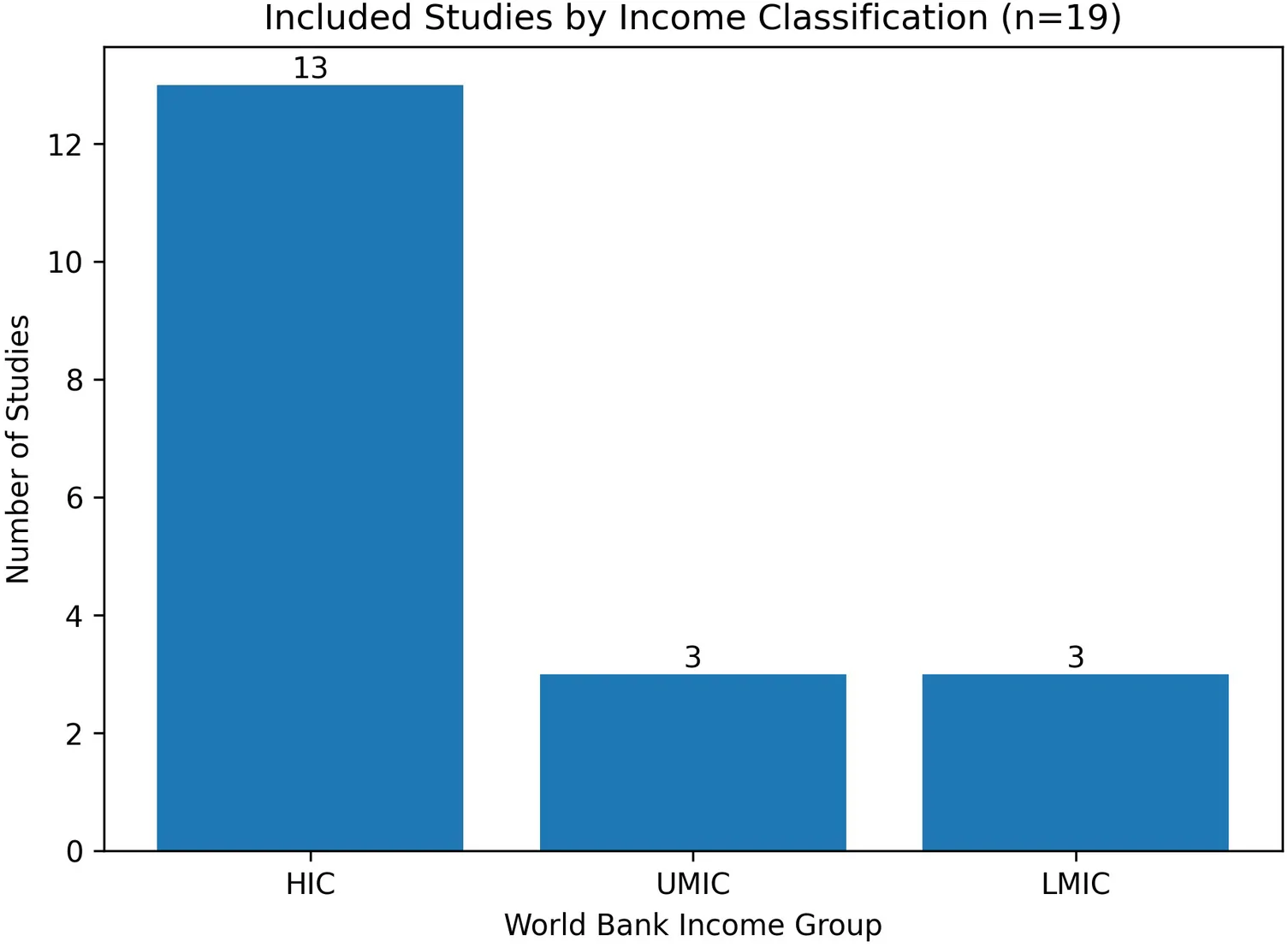
Distribution of included studies by World Bank income classification.

**Table 5:** Country contributions and income classification of included studies.

Country	Income	No. of studies	% of total
USA	HIC	5	26.3%
Sweden	HIC	2	10.5%
Japan	HIC	1	5.3%
Australia	HIC	1	5.3%
Denmark/Netherlands	HIC	1	5.3%
UK	HIC	1	5.3%
Germany	HIC	1	5.3%
Belgium	HIC	1	5.3%
China	UMIC	2	10.5%
Thailand	UMIC	1	5.3%
Brazil	LMIC	2	10.5%
Jordan	LMIC	1	5.3%

HIC, high-income country; UMIC, upper-middle-income country; LMIC, lower-middle-income country. Percentages based on n=19.

**Figure 3:**
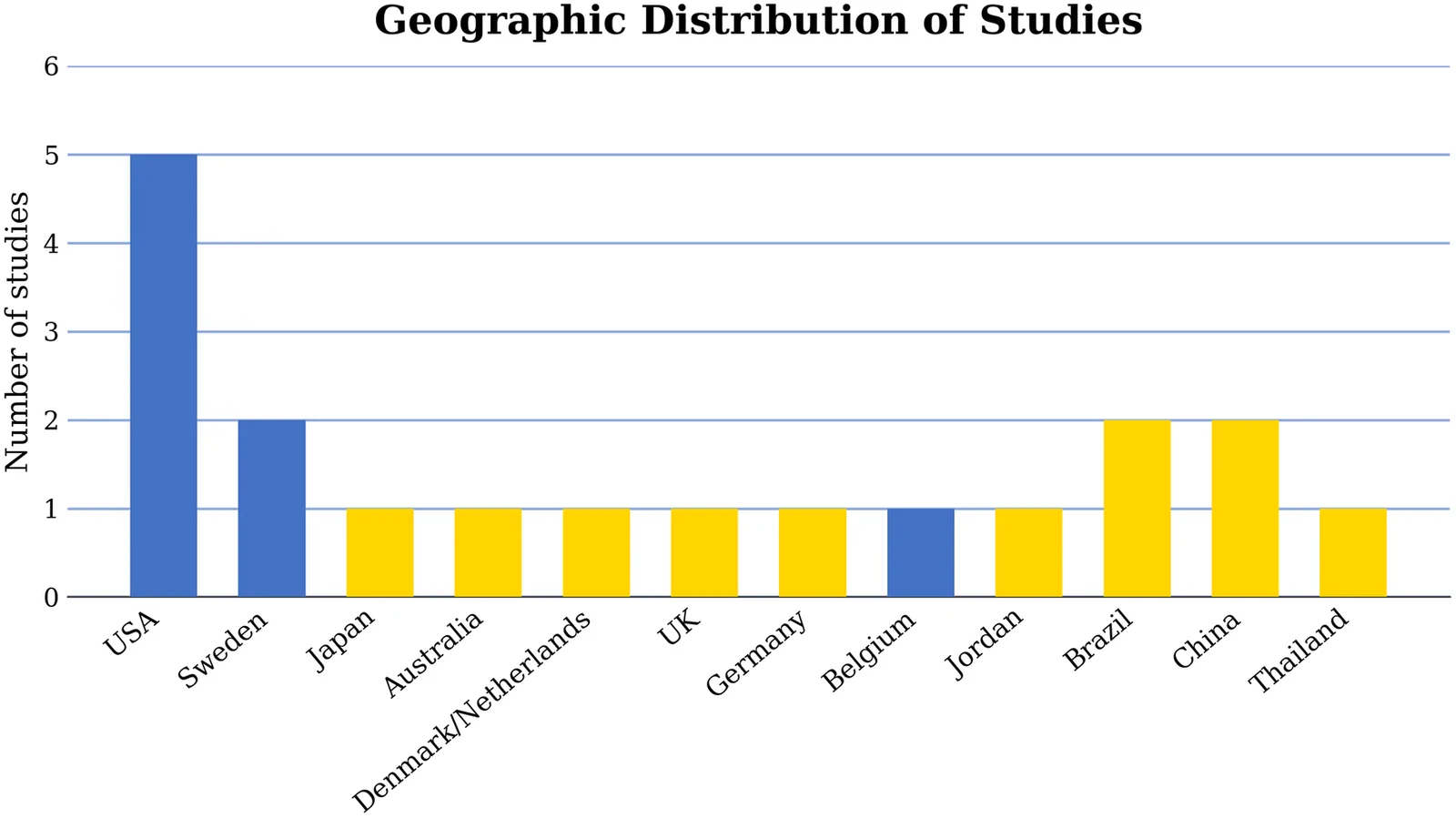
Geographic distribution of included studies and their income classification. This distribution reflects a concentration of included studies within high-income country settings, with limited representation from upper-middle- and lower-middle-income country contexts. All study types are presented together for mapping purposes only and do not imply equivalent levels of evidence.

#### 3.1.3 Study designs, sample sizes, and settings

Study designs ranged from RCTs and feasibility trials to observational and predictive ML studies (23, 46, 55, 56, 60). Sample sizes varied from small pilot cohorts (n=20) to large registry-based studies (n=7,638). Settings included schools, outpatient services, community platforms, and social media environments (33, 53, 54). A full study-level summary is provided in Supplementary Table S1 and evidence category classification in Supplementary Table S2.

#### 3.1.4 Reporting completeness and equity indicators

Equity-related reporting was inconsistent (Table 6 and Supplementary Table S3). Age was universally reported, while gender (57.9%), SES (10.5%), race/ethnicity (21.1%), and family involvement (21.1%) were limited. Cultural adaptation appeared in 42.1% of studies, primarily through translation or localization (33, 34, 36). Participatory co-design was rare, with the notable exception of REALbot (54). These gaps constrain generalizability and equity.

**Table 6:** Reporting completeness according to PROGRESS-plus equity indicators.

Reporting variable	No. of studies	Percentage
Age reported	19	100.0%
Gender reported	11	57.9%
Socioeconomic status (SES) measured	2	10.5%
Race/ethnicity reported	4	21.1%
Family involvement explicitly measured	4	21.1%
Cultural/linguistic adaptation reported	8	42.1%

Age was universally reported, whereas SES, race/ethnicity, and family-level variables were inconsistently documented (34, 55, 56). Cultural adaptation was typically limited to surface localization rather than participatory co-design, with few exceptions (54). These reporting gaps constrain generalizability and highlight the need for PROGRESS-Plus-aligned, equity-informed research designs.

### 3.2 RQ2: mental health domains

Domain coverage is summarized in Table 7 and Figure 4. Depression and anxiety dominated the literature (33, 34, 46, 53, 60), with short-term symptom improvements frequently reported. Secondary domains included body image, social functioning, stress regulation, and substance-use screening (35, 47, 56, 59). Trauma-related and eating-disorder interventions were absent.

**Table 7:** Summary of mental health domain representation.

Domain	n	Primary measures	Follow-up/notes
Depression & Anxiety	13	PHQ-9, GAD-7, GHQ-28	Short-term (≤2 mo); effects attenuated
Social anxiety	1	CSR, ADIS	≈3 mo
Body image/self-efficacy	1	Self-efficacy scales	72 h–1 mo
Social function	2	GHQ-28, Resilience	Short-term
Substance use	1	S2BI, BSTAD, TAPS	Clinic sample
Self-injury (NSSI)	1	DSHI-Y	Ongoing trial
Disruptive behavior	1	Aggression tasks	Pilot phase
Transdiagnostic/predictive	3	SDQ, LIWC, RNT	≤18 weeks
Stress & LLM Use	2	Stress subscales	Single time point
Minority well-being	1	PHQ-A, PROMIS	Post-pilot
PTSD	0		Gap in literature
Eating disorders	0		Gap in literature

CSR, Clinical Severity Rating; ADIS, Anxiety Disorders Interview Schedule; SDQ, Strengths and Difficulties Questionnaire; RNT, Repetitive Negative Thinking.

**Figure 4:**
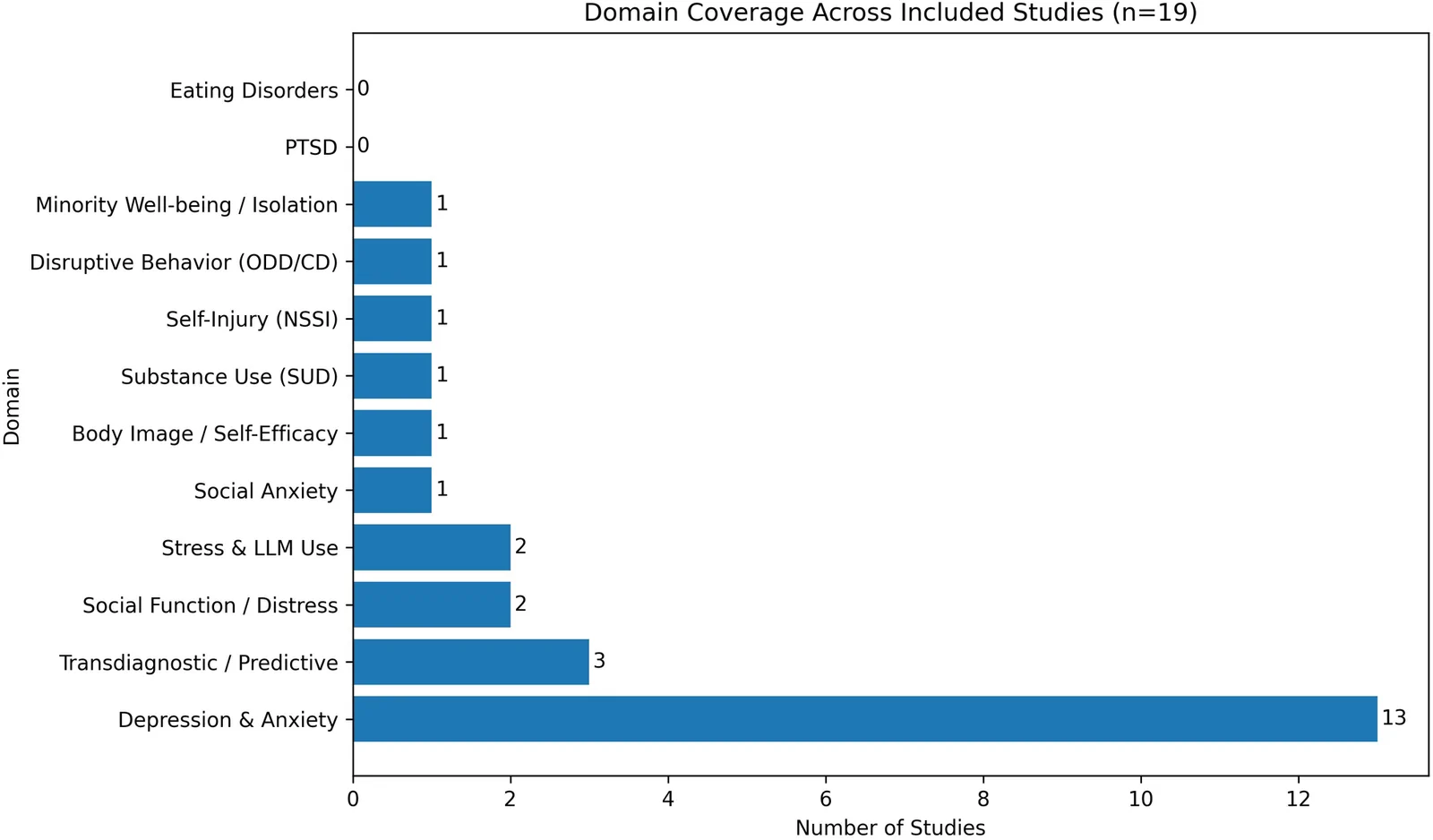
Mental health domains addressed across included studies (n=19).

#### 3.2.1 Depression and anxiety

Short-term improvements were observed across CBT apps, therapist-guided iCBT, and chatbot-supported interventions (23, 46, 60). Predictive ML models and speech-based phenotyping approaches demonstrated technical feasibility for early detection (55, 58, 61), though long-term durability of symptom improvements remains uncertain.

#### 3.2.2 Emerging and secondary domains

Emerging research addressed body image, self-efficacy, behavioral regulation, and stress monitoring (35, 52, 59). Evidence remains fragmented, indicating early-stage diversification. Depression and anxiety were the predominant focus areas, whereas trauma-related and eating disorder interventions were absent. These patterns provide context for the descriptive coded-theme distribution presented in Section 3.3.

### 3.3 RQ3: distribution of coded themes within the mapped corpus (2020–2025)

Figure 5 summarizes coded-theme frequency by year for digital mental health interventions for adolescents and youth between 2020 and 2025; given the small number of included studies (n=19), this is presented as a descriptive frequency count rather than a statistically supported trend. The coded themes varied across the included studies and years, with feasibility and therapist-supported digital CBT represented among earlier studies, while later studies in the corpus included digital phenotyping, LLM-based screening, governance-oriented frameworks, and culturally adaptive intervention designs (23, 34, 36, 49, 55, 56, 60). These observations describe the distribution of coded themes within the included corpus and do not imply temporal evolution, causal progression, or field-wide research trend.

**Figure 5:**
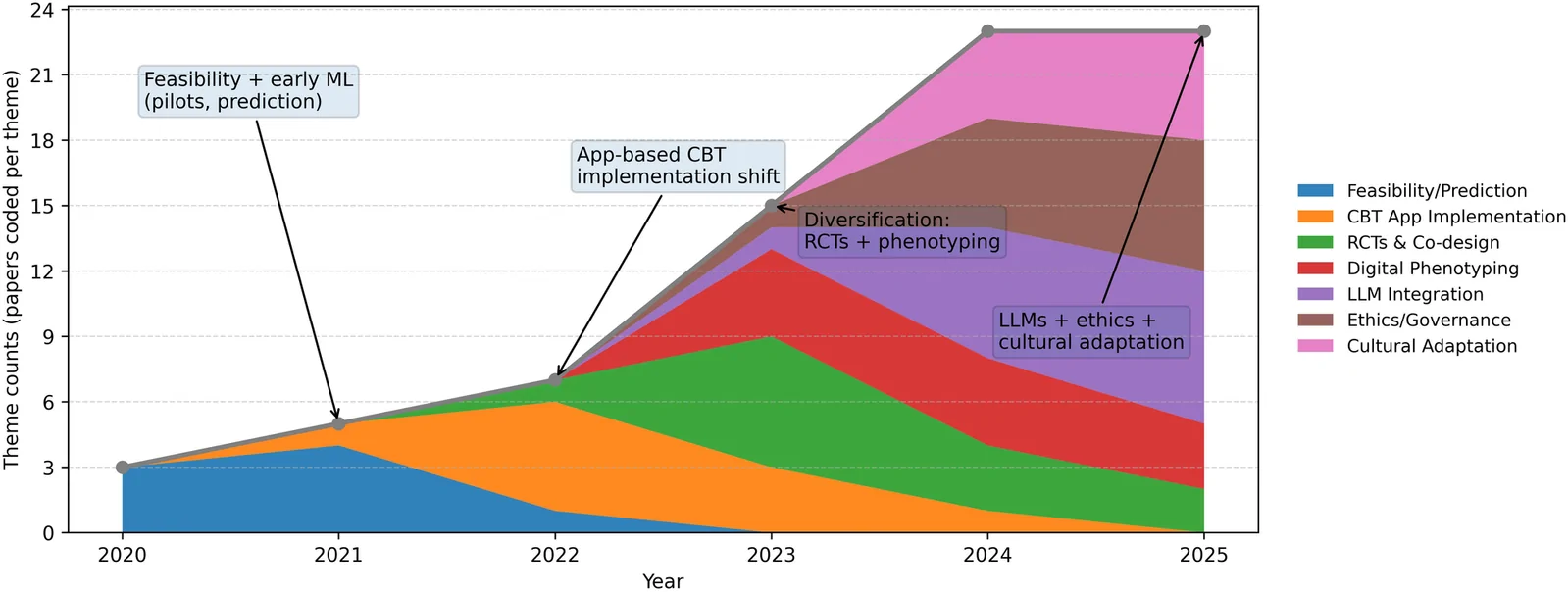
Coded-theme frequency in digital adolescent mental health research (2020–2025), by year. Stacked areas represent coded-theme frequency only, not a temporal trend or effect estimate; individual studies may contribute to multiple themes across multiple years, so category totals exceed the underlying study count of 19. The overlaid line indicates the total number of coded studies per year. Given the small number of included studies (n=19) distributed unevenly across five years, this figure should be read descriptively and not as evidence of a statistically meaningful trend.

### 3.4 RQ4: digital interventions and AI chatbots

A conceptually important distinction must be established before presenting the intervention typology. Therapist-guided internet-delivered CBT (iCBT) platforms are digitally mediated but rely entirely on human-authored structured content and clinician oversight; they contain no meaningful AI component and function as human-led digital delivery systems. In contrast, AI-enabled tools including rule-based chatbots, hybrid conversational agents, LLM-based systems, and predictive ML models incorporate automated, data-driven processing to generate responses, identify risk patterns, or adapt content dynamically. Conceptual governance papers constitute a third distinct category. Non-AI digital interventions using structured or rule-based logic were retained as comparator evidence and were analyzed separately from AI-enabled tools. Throughout this section, these three categories are treated separately. Claims regarding AI chatbot evidence apply exclusively to AI-enabled tools and do not extend to iCBT platforms or governance frameworks.

Intervention typologies are summarized in Supplementary Table S4 and Figure 6. Key findings across intervention types included:

1. **Therapist-guided iCBT modules** targeting depression and anxiety, typically delivered through structured internet-based programmes with clinician involvement (23, 46).
2. **Smartphone-based CBT and Just-in-Time Adaptive Interventions (JITAIs)** focusing on real-time mood regulation and repetitive negative thinking through adaptive mobile platforms (60).
3. **Structured emotion-regulation and preventive tools** incorporating behavioral activation, psychoeducation, and self-monitoring components (57, 59).
4. **AI-based screening and conversational tools** supporting automated assessment or conversational interaction in the included studies (36, 47).
5. **Predictive and speech-derived phenotyping frameworks** utilizing ML techniques for early risk identification and monitoring (55, 58).

The included studies illustrate variation in how these approaches were combined, including hybrid digital ecosystems incorporating multiple intervention or assessment functions. These observations describe the characteristics of the included corpus and should not be interpreted as evidence of a temporal increase or field-wide transition toward more integrated systems.

**Figure 6:**
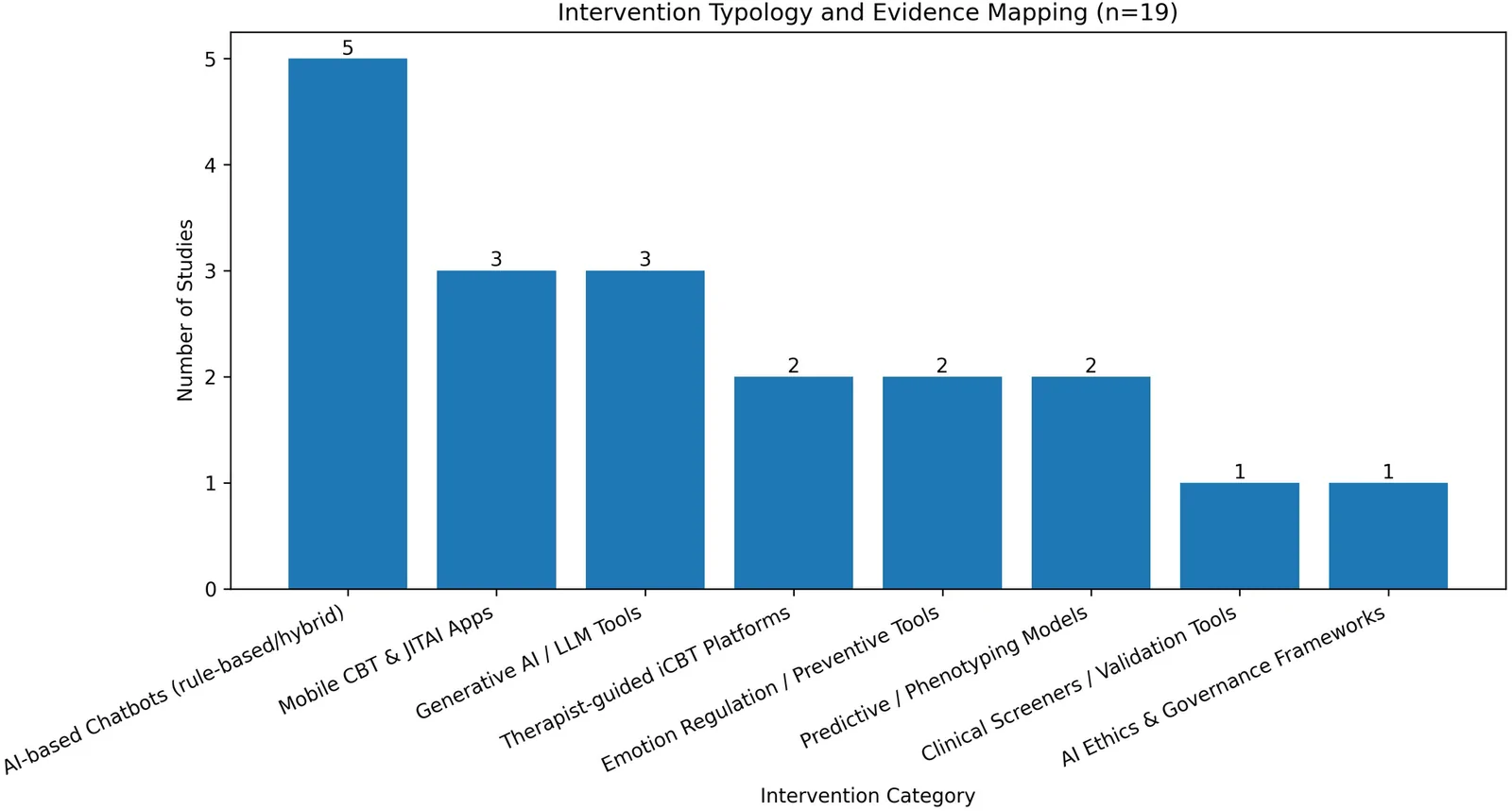
Study design distribution and intervention typology across included studies (n=19). The vertical axis reflects study design characteristics as reported by authors and does not represent a quality hierarchy or risk-of-bias assessment. Positioning is based solely on descriptive study design categorization consistent with scoping review methodology (41).

#### 3.4.1 Characteristics of digital interventions across the included corpus

The included studies varied in methodological and technological characteristics across the review period. Studies published earlier in the corpus included predictive ML modeling and therapist-directed iCBT, while later studies included app-based CBT, digital phenotyping, LLM-based screening, governance frameworks, and hybrid conversational systems. These observations describe the characteristics of the included corpus and should not be interpreted as evidence of temporal progression or a field-wide developmental trajectory (23, 33, 35, 36, 46, 49, 52, 55–57, 59, 60).

### 3.5 Limitations of AI-based tools

Key limitations are summarized in Supplementary Table S5. These include methodological constraints (55, 58), engagement decline (60), bias risks (56), ethical and legal concerns (49), and limited adolescent-specific RCT evidence (33, 34). Studies conducted in Sweden and Japan established early evidence using predictive ML (55) and therapist-directed iCBT (23). Follow-up research led by Australia, Denmark, and Germany (52, 57, 60) expanded the field through applied trials on mobile CBT, emotion regulation, and feasibility of digital delivery.

Studies examining chatbots such as Vitalk (33), Elomia combined with GymBuddy (35), and GPT-based screeners reflect growing interest in semi-automated and generative approaches. We note that the Vitalk evaluation (33) was authored by study-affiliated developers of the commercial product and drew on a general-population sample that was 52% aged 18–24 years and 76% female, a design and sampling profile that should be weighed when interpreting its findings alongside the randomized evidence included elsewhere in this review. These characteristics should be considered when interpreting the generalizability of the reported findings to adolescent-specific mental health settings. Regionally localized studies (34, 47) and the governance-focused analysis by Adesina et al. (49) reflect research efforts directed at transparency and ethical oversight. Interventions most frequently examined in the literature remain therapist-guided CBT formats, whereas AI-enabled chatbots, predictive models, and LLM-based screening systems represent an emerging but methodologically heterogeneous evidence base.

## 4 Discussion

This review maps the current landscape of AI-enabled and digital mental health interventions for adolescents and youth as represented in 19 peer-reviewed studies published between 2020 and 2025. The findings identify patterns in research focus, intervention types, and reporting practices, alongside persistent gaps in clinical validation, cultural adaptation, and ethical governance. Given the limited number of included studies, substantial heterogeneity in study designs, uneven geographical distribution, and the absence of formal risk-of-bias assessment, all findings should be interpreted as descriptive mapping rather than evidence of effectiveness, maturity, or field-level consensus, consistent with PRISMA-ScR methodology (41).

### 4.1 Characteristics of the mapped evidence base

Across the included studies, AI-based approaches included chatbot-assisted screening, LLM-supported assessment, predictive modeling, and conversational interventions, alongside therapist-guided digital CBT (36, 55, 58). The distribution of these approaches reflects the heterogeneity of the included evidence rather than a demonstrated field-wide transition. Given the small number of studies and their methodological and contextual heterogeneity, temporal or developmental conclusions cannot be reliably drawn from the mapped corpus alone.

Examining the mapped evidence through the Human–AI Collaboration Role framework reveals a descriptive pattern that merits attention. Study-level classifications for all 19 included studies are provided in Supplementary Table S7. Of the 19 included studies, the majority of intervention and feasibility studies (n=11) involved either Level 1 (No AI human-led digital delivery) or Level 3 (AI-primary) tools, with the strongest controlled evidence concentrated at Level 1. Studies involving Level 3 (AI-primary) tools were predominantly observational, feasibility, or quasi-experimental in design, with limited randomized evidence. No included study provided evidence sufficient to establish clinical equivalence or superiority for a system operating without human oversight in this population. This distribution indicates that across the mapped corpus, higher levels of AI autonomy are associated with weaker study designs and more limited clinical evidence, a pattern with direct implications for how AI-enabled tools are positioned within stepped-care frameworks for adolescents. This association should not be read as evidence that more autonomous systems are inherently unsuitable; rather, it highlights a gap in the evidence base itself, since higher-autonomy tools have simply not yet been evaluated using the more rigorous designs applied to lower-autonomy interventions in this mapped corpus. Closing this gap through adequately powered controlled trials, as recommended in Section 4.10, is necessary before the comparative safety and effectiveness of higher-autonomy systems can be established, rather than assumed from the current evidence distribution alone.

### 4.2 Clinical value and effectiveness

Before discussing clinical findings, it is important to note that two included studies Samatha et al. (47) and Vanhoffelen et al. (48) are classified as user-perception studies rather than intervention and feasibility studies. These cross-sectional surveys report adolescents’ attitudes toward and emotional reactions to general AI tools; they do not deliver or evaluate a clinical intervention and are not cited in support of any clinical outcome claim in this section. All clinical observations below apply exclusively to the 11 intervention and feasibility studies and 4 AI screening and validation studies. The four AI screening and validation studies (36, 55, 56, 58) are considered separately for technical and screening-performance evidence and are not treated as clinical intervention and feasibility studies.

Within the included studies, AI applications were predominantly evaluated as supportive tools within hybrid or human-supervised care models rather than as replacements for human-delivered therapy (53, 54). Some intervention studies reported short-term symptom improvements, but the durability of these effects remains uncertain (46, 60). It is important to state precisely what the evidence does and does not show for participants under 18 years: no fully randomized diagnostic chatbot trials were identified in this age group (Supplementary Table S5), but this does not extend to chatbot-delivered interventions more broadly. Matheson et al. (59), for example, is a two-arm randomized controlled trial of a chatbot-delivered body-image intervention conducted in participants aged 13–18. Comparative claims about intervention outcomes should therefore be made only when applying to the specific study design and clinical purpose in question, rather than to chatbot-based interventions for minors as a whole.

### 4.3 Methodological patterns in youth AI chatbot research

Across the included studies, common methodological features included small sample sizes, short follow-up durations, and inconsistency in outcome measurement tools. Few studies examined integration with clinical supervision models or assessed long-term outcomes, indicating that further methodological development is needed before robust clinical conclusions can be drawn.

### 4.4 Comparison with prior reviews

Prior evaluations have produced significant data about digital and AI-driven mental health interventions but their scope and analytical emphasis diverge from the current study. Borghouts et al. (19) compiled barriers and facilitators of user engagement across digital mental health interventions, yielding insights into usability and contextual implementation factors, yet lacking a concentrated emphasis on AI-driven conversational agents, early detection mechanisms, or adolescent demographics. Li et al. (62) and He et al. (63) conducted systematic reviews and meta-analyses demonstrating that conversational agents can produce short-term reductions in depression and anxiety, with findings emphasizing the importance of personalization and empathetic system responses. Nonetheless, these syntheses mostly focused on treatment efficacy in adult populations.

The present scoping review maps AI-driven digital mental health tools for adolescents and youth, with attention to early detection, intervention design characteristics, and ethical considerations. By including study types beyond RCTs, this review identifies gaps in safety, regulatory oversight, cultural adaptation, and youth-specific safeguarding that are underrepresented in prior effectiveness-focused syntheses.

### 4.5 Clinical integration gap

Most AI-enabled interventions were evaluated as standalone tools rather than embedded components within stepped-care pathways or clinician-supervised treatment frameworks. Limited evidence assessed how digital tools interact with therapist workflows, referral systems, or school-based mental health services. Bridging this integration gap is critical for sustainable adoption.

### 4.6 Cultural and domain considerations

Current interventions overemphasize depression and anxiety while underrepresenting PTSD, substance use, and loneliness, consistent with patterns reported in prior digital mental health reviews (19, 21). Cultural adaptation remains limited, with few studies incorporating linguistic localization or culturally grounded coping frameworks (33, 34, 54). This constrains ecological validity and long-term engagement, particularly in LMIC contexts where sociocultural determinants of mental health differ significantly (3).

These equity reporting gaps carry substantive implications. Three interconnected concerns merit closer examination.

**External validity.** The predominance of studies conducted in HICs (68.4%) means that the mapped evidence base reflects the mental health contexts, help-seeking behaviors, and technological infrastructure of a narrow demographic. When cultural adaptation is limited to surface localization or translation of existing content, without restructuring underlying assumptions about symptom expression, family dynamics, or stigma the resulting tools may measure and respond to psychological distress in ways poorly calibrated for non-Western populations. The absence of participatory co-design in all but one included study (54) further limits ecological validity.

**Algorithmic bias.** The insufficient reporting of race/ethnicity (21.1% of studies) and SES (10.5% of studies) has direct implications for algorithmic fairness. ML-based screening tools and predictive models trained predominantly on data from high-income, predominantly White or East-Asian adolescent cohorts may systematically underperform or misclassify adolescents from underrepresented ethnic, socioeconomic, or linguistic backgrounds. Speech-based phenotyping models (58) are particularly vulnerable to dataset shift when applied across cultural and linguistic boundaries. LLM-based screening tools (36) trained on predominantly English-language clinical data may embed culturally specific assumptions that do not transfer to non-Western adolescent populations.

**Applicability in diverse social and cultural contexts.** Digital mental health tools designed and validated in high-income settings face compounding barriers to applicability in LMIC and culturally diverse contexts. Socioeconomic factors including smartphone ownership, reliable internet access, digital literacy, and data costs are rarely assessed despite their direct relevance to scalability. Family-level variables, reported in only 21.1% of included studies, are particularly important in collectivist cultural contexts where family involvement in adolescent health decisions is normative.

Taken together, these three dimensions external validity, algorithmic bias, and cross-cultural applicability indicate that the equity gaps identified in this review are structural limitations with direct consequences for the safe and equitable deployment of AI-enabled mental health tools. Future research should prioritize PROGRESS-Plus-aligned data collection, culturally grounded co-design processes, and systematic bias auditing of AI models before deployment in diverse adolescent populations.

### 4.7 Safety and safeguarding for minors

Given the vulnerability of adolescent and youth populations, safeguarding considerations are central to the deployment of AI-based mental health systems. As noted in Section 3.1, only 4 of the 19 included studies enrolled participants exclusively under 18 years, while 9 studies enrolled samples spanning both minor and adult participants without age-disaggregated outcomes; safeguarding claims in this section should therefore be read with this demographic composition in mind. Across the reviewed studies, crisis detection mechanisms such as identifying self-harm ideation, severe distress, or rapid symptom escalation were inconsistently implemented and rarely standardized (Supplementary Table S6). While some systems incorporated predefined risk thresholds or keyword-based triggers, comprehensive escalation pathways were often underreported.

Effective safeguarding requires clearly defined escalation protocols that specify when and how alerts are transferred to clinicians, caregivers, or emergency services. Most reviewed systems relied on passive recommendations rather than enforceable referral mechanisms, raising concerns about delayed intervention during high-risk episodes. Moreover, clinician and guardian involvement was frequently optional rather than structurally embedded.

Fully autonomous decision-making carries elevated risk in high-risk mental health contexts involving youth given the current evidence base, and human-in-the-loop oversight for diagnostic interpretation, crisis management, and care planning represents the more cautious approach available today. Whether fully autonomous systems could be appropriate in specific circumstances, such as expanding access to mental health support in underserved communities where human-delivered care is otherwise unavailable, remains an open empirical question rather than a settled one, since no included study in this review directly tested fully automated systems against human-in-the-loop alternatives in controlled conditions. Auditability and traceability of system decisions particularly for risk classification and escalation events remain critical gaps, underscoring the need for transparent logging, review mechanisms, and governance frameworks tailored to minor populations.

### 4.8 Risks and challenges of generative AI systems

The recent integration of large language models (LLMs) into digital mental health applications introduces both opportunities and distinct risks (64). One key concern is hallucination, whereby generative systems may produce inaccurate, misleading, or clinically inappropriate responses, particularly in emotionally sensitive contexts (64). In adolescent populations, such responses risk undermining trust or providing harmful guidance if not adequately constrained.

Over-trust in conversational agents represents another challenge, as youth users may anthropomorphize AI systems or attribute clinical authority to them. Privacy leakage is also a critical issue, given the potential for sensitive mental health data to be exposed through prompt logging, model training artifacts, or unintended data retention (64). Non-determinism and limited reproducibility complicate evaluation, especially when model outputs vary across interactions. These limitations highlight the need for rigorous, safety-oriented evaluation frameworks before deploying generative AI systems in youth mental health contexts.

### 4.9 Implementation and adoption considerations

Beyond technical performance, the real-world impact of AI-based mental health systems depends on successful implementation within existing care ecosystems (65). Several studies reported increased workflow burden for clinicians, particularly when AI outputs required manual verification or duplicate documentation, consistent with broader implementation research showing that compatibility with existing clinical workflows is a key determinant of digital mental health tool adoption (65). Without seamless integration into clinical information systems, such tools risk remaining adjunctive additions to care rather than meaningfully integrated components of it.

School-based deployment presents additional challenges, including coordination with educational staff, alignment with safeguarding policies, and access to referral networks. Many reviewed systems lacked clearly defined downstream pathways, raising concerns about referral readiness and continuity of care. Stakeholder acceptance including clinicians, educators, caregivers, and adolescents themselves emerged as a critical determinant of adoption.

### 4.10 Implications and future directions

The following priorities are suggested based on descriptive patterns observed across the 19 included studies and the gaps identified through systematic mapping. Five descriptive gap-based priorities emerge from this mapping:

1. **Clinical validation:** Conduct adequately powered RCTs with longitudinal follow-up to confirm both short- and long-term efficacy, retention, and durability of AI-enabled mental health tools.
2. **Participatory co-design:** Involve adolescents, families, and clinicians as co-creators throughout the design and evaluation process to ensure inclusivity, cultural relevance, and contextual fit (54).
3. **Clinical integration:** Embed AI and digital tools within existing care pathways and referral networks, systematically evaluating their impact on therapist workload, care continuity, and sustained engagement (53).
4. **Ethical and regulatory alignment:** Establish clear frameworks governing informed consent, data privacy, and crisis escalation procedures proportional to the degree of AI autonomy and user vulnerability (49).
5. **Sustained engagement:** Design hybrid systems that balance automation with human oversight to preserve empathy, trust, and adherence over extended periods of use.

Future research would benefit from greater emphasis on systemic integration within clinical and educational infrastructures, alongside culturally adaptive design and transparent ethical governance frameworks.

### 4.11 Terminological inconsistency in the field

A recurring challenge encountered during screening and data charting was terminological inconsistency across the included literature. Terms such as “AI chatbot,” “conversational agent,” “virtual assistant,” and “digital mental health tool” were used interchangeably across studies without consistent operational definitions, complicating classification and comparison. This pattern mirrors observations in adjacent fields where emerging technologies outpace standardization of vocabulary (41). The operational definitions established prior to full review (Table 1) represent a deliberate response to this gap, applied consistently across all screening and data charting stages. Standardization of terminology in this rapidly evolving field would substantially improve the coherence and comparability of future research syntheses.

### 4.12 Strengths of the review

This review integrates AI chatbots, digital screening tools, and ethical governance within a youth-specific framework, which remains underrepresented in prior syntheses. Unlike prior scoping reviews in this domain that drew primarily on theoretical, editorial, or commentary sources, the present review includes 11 intervention and feasibility studies and 4 AI screening and validation studies, providing a stronger empirical foundation for descriptive mapping. Use of PRISMA-ScR ensured transparent mapping, protocol registration on OSF (created prior to the database search and subsequently formalized as a DOI-assigned record) supported methodological pre-specification, and inclusion of LMIC studies improves global relevance. The Human–AI Collaboration Role framework (developed under the working title “Human–AI Symmetry” in the OSF project and subsequently relabeled “Human–AI Collaboration Role” during manuscript preparation to clarify its functional focus) was applied as a predefined coding dimension as an analytic contribution adapted from prior automation-level frameworks (26, 29), enabling systematic classification of intervention types across the included corpus.

### 4.13 Limitations

This review has several limitations. Restriction to English-language publications is a substantive limitation given this review’s stated emphasis on cultural adaptation and equity: relevant regional research published in other languages, particularly from LMIC and non-Western contexts, may have been systematically excluded, potentially biasing the mapped evidence base further toward the high-income, English-language settings already shown to dominate the corpus (68.4% HIC; see Section 3.1). This constraint should be considered when interpreting the equity and cultural-adaptation findings reported throughout this review. We further note, in the interest of full transparency, that no frozen, timestamped registration existed prior to or during either the primary (November 2025) or supplementary (January 2026) searches; the DOI-assigned OSF registration (10.17605/OSF.IO/SBPK2) was completed on 11 March 2026, after all data collection had concluded. We therefore do not characterize this review as predefined registered in the formal sense. As a scoping review, no formal risk-of-bias appraisal or meta-analytic synthesis was conducted, consistent with PRISMA-ScR guidance. The rapid pace of AI development means that new tools may have emerged beyond the review timeframe, and some included systems may have been updated or discontinued since publication. Reliance on published literature may underrepresent real-world implementations described in grey or industry sources. Additional limitations include underrepresentation of LMICs, inconsistent reporting of engagement metrics, and heterogeneity in outcome measures across studies.

## 5 Conclusion

This PRISMA-ScR scoping review maps AI chatbots, digital screening tools, and CBT-based interventions for adolescent and youth mental health (10–24 years) published between 2020 and 2025. Across the 19 included studies, therapist-guided and structured CBT approaches were the most frequently studied using randomized and controlled designs. AI chatbots, predictive models, and LLM-based systems have been explored in a smaller number of studies, with feasibility or engagement outcomes reported in a limited number of studies and contexts.

The current evidence base is largely concentrated on depression and anxiety, with limited attention to trauma-related, eating, and other underrepresented mental health conditions. Moreover, inconsistent reporting of equity, cultural adaptation, and implementation variables reveals significant gaps in the literature. Key challenges include safeguarding for minors, privacy and consent, algorithmic bias, and insufficient integration with clinical and educational systems.

Future research should prioritize adequately powered adolescent-focused trials, participatory co-design approaches, culturally informed adaptations, and transparent governance frameworks. Enhancing human-in-the-loop mechanisms and strengthening real-world implementation pathways will be essential to ensure that AI-enabled mental health tools are developed and evaluated in ways that are safe, contextually appropriate, and responsive to the needs of diverse adolescent populations. Overall, this review identifies persistent gaps in clinical validation, cultural adaptation, and ethical governance across the mapped evidence base, and underscores the need for rigorous, context-sensitive, and equity-informed research to guide the responsible development of digital mental health tools for adolescents and youth. We note that this review’s restriction to English-language publications may have systematically excluded relevant evidence from non-Western and low- and middle-income contexts, a constraint that should be weighed directly against the equity and cultural-adaptation conclusions drawn above.

## Data Availability

The original contributions presented in the study are included in the article/Supplementary Material, further inquiries can be directed to the corresponding authors.
