# Supplementary material for: AI-enabled and comparator digital interventions for adolescent mental health (2020–2025): early detection, human–AI collaboration, and ethical governance—a PRISMA-ScR scoping review
Source: Front Child Adolesc Psychiatry. 2026 Sep 30;5:1905782. doi: 10.3389/frcha.2026.1905782 (PMC13661949; doi:10.3389/frcha.2026.1905782)
Supplement: Supplementary file 1 [file Datasheet1.pdf]

Supplementary files on database searching Strategies.

PubMed (n = 837) (R) ALL <2020 to November, 2025>		
1	(adolescent* OR youth OR "young people" OR teen* OR "young adult*")	
2	("mental health"[tiab]<br>OR "digital mental health"[tiab]<br>OR depression[tiab]<br>OR anxiety[tiab]<br>OR stress[tiab])	
3	(chatbot*[tiab]<br>OR "conversational agent*"[tiab]<br>OR "artificial intelligence"[tiab]<br>OR AI[tiab]<br>OR "machine learning"[tiab]<br>OR "deep learning"[tiab]<br>OR "large language model*"[tiab]<br>OR "generative AI"[tiab])	
4	1 AND 2 AND 3	837

Scopus (n = 2365) (R) ALL <2020 to November, 2025>		
1	TITLE-ABS-KEY (adolescent* OR youth OR "young people" OR teen* OR "young adult*")	
2	TITLE-ABS-KEY ("mental health" OR "digital mental health" OR depression OR anxiety OR stress)	
3	TITLE-ABS-KEY (chatbot* OR "conversational agent*" OR "artificial intelligence" OR AI<br>OR "machine learning" OR "deep learning"<br>OR "large language model*" OR "generative AI")	
4	1 AND 2 AND 3	2365

Web of Science (n = 1607) (R) ALL <2020 to November, 2025>		
1	TS=(adolescent* OR youth OR "young people" OR teen* OR "young adult*")	
2	TS=("mental health" OR "digital mental health" OR depression OR anxiety OR stress)	
3	TS=(chatbot* OR "conversational agent*" OR "artificial intelligence" OR AI<br>OR "machine learning" OR "deep learning"<br>OR "large language model*" OR "generative AI")	
4	1 AND 2 AND 3	1607

Supplementary files on database searching Strategies.

Springer Nature Link (n = 2249) (R) ALL <2020 to November, 2025>		
1	(adolescent* OR youth OR "young people" OR teen* OR "young adult*")	
2	("mental health" OR "digital mental health" OR depression OR anxiety OR stress)	
3	(chatbot* OR "conversational agent*" OR "artificial intelligence" OR AI OR "machine learning" OR "deep learning" OR "large language model*" OR "generative AI")	
4	1 AND 2 AND 3	2249

ProQuest (n = 2149) (R) ALL <2020 to November, 2025>		
1	(adolescent* OR youth OR "young people" OR teen* OR "young adult*")	
2	("mental health" OR "digital mental health" OR depression OR anxiety OR stress)	
3	(chatbot* OR "conversational agent*" OR "artificial intelligence" OR AI OR "machine learning" OR "deep learning" OR "large language model*" OR "generative AI")	
4	1 AND 2 AND 3	2149

ScienceDirect (n = 1755) (R) ALL <2020 to November, 2025>		
1	adolescent OR youth OR "young people" OR teen OR "young adult"	
2	"mental health" OR "digital mental health" OR depression OR anxiety OR stress	
3	chatbot OR "conversational agent" OR "artificial intelligence" OR "machine learning" OR "large language model" OR "generative AI"	
4	1 AND 2 AND 3	1755
